# Supplementary material for: The quantity and composition of household food waste: Implications for policy
Source: PLoS One. 2024 Jun 12;19(6):e0305087. doi: 10.1371/journal.pone.0305087 (PMC11168659; doi:10.1371/journal.pone.0305087)
Supplement: S1 Table — (DOCX) [file pone.0305087.s001.docx]

Supplementary Table 1 Mean and standard deviation of FW quantity by type and food group in urban and rural areas (kg/cap/year)

| **Food Groups** | **Urban** | | | **Rural** | | | | **Edible FW**  **(urban-rural)** | | **Inedible FW**  **(urban-rural)** | | **Total FW**  **(urban-rural)** | |
| --- | --- | --- | --- | --- | --- | --- | --- | --- | --- | --- | --- | --- | --- |
|  | **Edible FW** | **Inedible FW** | **Total FW** | **Edible FW** | **Inedible FW** | **Total FW** | **p** | | **CI 95%** | **p** | **CI 95%** | **p** | **CI 95%** |
| Cereals, tubers and their derivative products | 17.9 ± 4.9 | 3 ± 0.8 | 20.9 ± 5.8 | 3.5 ± 1 | 3 ± 0.8 | 6.5 ± 1.8 | <0.001 | | 13.6-15.3 | 0.714 | -0.3-0.2 | <0.001 | 13.3-15.4 |
| Bakery products | 1.1 ± 0.3 | - | 1.1 ± 0.3 | 0.4 ± 0.1 | - | 0.4 ± 0.1 | <0.001 | | 0.7-0.8 | - | - | <0.001 | 0.7-0.8 |
| Meat and meat products | 0.2 ± 0.1 | 2.1 ± 0.6 | 2.3 ± 0.6 | 0 ± 0 | 1 ± 0.3 | 1 ± 0.3 | <0.001 | | 0.2-0.2 | <0.001 | 0.9-1.2 | <0.001 | 1.1-1.3 |
| Fish and fishery products | 0.2 ± 0.1 | 1.3 ± 0.3 | 1.5 ± 0.4 | 0.1 ± 0 | 0.2 ± 0 | 0.3 ± 0.1 | <0.001 | | 0.1-0.1 | <0.001 | 1-1.1 | <0.001 | 1.1-1.3 |
| Eggs and dairy products | 0.1 ± 0 | 2 ± 0.5 | 2.1 ± 0.6 | 0 ± 0 | 0.9 ± 0.2 | 0.9 ± 0.2 | <0.001 | | 0.1-0.1 | <0.001 | 1-1.2 | <0.001 | 1.1-1.3 |
| Spices | 0.7 ± 0.2 | 1.8 ± 0.5 | 2.5 ± 0.7 | 0.3 ± 0.1 | 0.8 ± 0.2 | 1.1 ± 0.3 | <0.001 | | 0.4-0.4 | <0.001 | 0.9-1.1 | <0.001 | 1.3-1.6 |
| Legumes | 1.3 ± 0.3 | 0.6 ± 0.2 | 1.9 ± 0.5 | 0.2 ± 0.1 | 0.9 ± 0.3 | 1.2 ± 0.3 | <0.001 | | 1-1.1 | <0.001 | -0.4--0.2 | <0.001 | 0.6-0.8 |
| Vegetables | 6.2 ± 1.7 | 8.3 ± 2.3 | 14.4 ± 4 | 4.6 ± 1.3 | 6.6 ± 1.8 | 11.2 ± 3.1 | <0.001 | | 1.1-2 | <0.001 | 1-2.3 | <0.001 | 2.1-4.3 |
| Fruit and derivative products | 1.1 ± 0.3 | 29.9 ± 8.3 | 31 ± 8.6 | 1 ± 0.3 | 21.7 ± 6 | 22.7 ± 6.3 | 0.424 | | -0.1-0.1 | <0.001 | 6.3-10.3 | <0.001 | 6.3-10.4 |
| Candy and chocolate | 0 ± 0 | - | 0 ± 0 | 0 ± 0 | - | 0 ± 0 | <0.001 | | 0-0 | - | - | <0.001 | 0-0 |
| Mixed foods/snacks | 0.9 ± 0.2 | - | 0.9 ± 0.2 | 0.2 ± 0.1 | - | 0.2 ± 0.1 | <0.001 | | 0.7-0.7 | - | - | <0.001 | 0.7-0.7 |
| Other foods | - | 0.1 ± 0 | 0.1 ± 0 | - | 0 ± 0 | 0 ± 0 | <0.001 | | - | <0.001 | 0.1-0.1 | <0.001 | 0.1-0.1 |
| Drinks | 0.7 ± 0.2 | - | 0.7 ± 0.2 | 0.3 ± 0.1 | - | 0.3 ± 0.1 | <0.001 | | 0.4-0.4 | - | - | <0.001 | 0.4-0.4 |
| **Total** | **30.3 ± 8.4** | **49.1 ± 13.5** | **79.4 ± 21.9** | **10.7 ± 3** | **35.1 ± 9.7** | **45.8 ± 12.6** | **<0.001** | | **18.1-21.1** | **<0.001** | **10.7-17.2** | **<0.001** | **28.8-38.2** |
