# Supplementary material for: The quantity and composition of household food waste: Implications for policy
Source: PLoS One. 2024 Jun 12;19(6):e0305087. doi: 10.1371/journal.pone.0305087 (PMC11168659; doi:10.1371/journal.pone.0305087)
Supplement: S2 Table — (DOCX) [file pone.0305087.s002.docx]

Supplementary Table 1 Quantity of frequent edible food waste (mean g/cap/year)

| **Foods** | **Urban** | **Rural** | **Total** |
| --- | --- | --- | --- |
| Cereals, tubers and their derivative products |  |  |  |
| Rice | 15,197.8 | 1,413.7 | 14,228.7 |
| Noodles | 872.4 | 221.0 | 826.6 |
| Potato | 490.2 | 319.4 | 478.2 |
| Casava | 380.0 | 192.9 | 366.8 |
| Sweet potato | 278.1 | 786.0 | 313.8 |
| Taro | 137.8 | 283.3 | 148.0 |
| Vegetables |  |  |  |
| Carrot | 973.4 | 429.9 | 935.2 |
| Chinese cabbage | 766.9 | 279.4 | 732.6 |
| Cabbage | 629.4 | 401.2 | 613.3 |
| Cucumber | 508.6 | 212.3 | 487.7 |
| Water spinach | 353.4 | 306.6 | 350.1 |
| Long beans | 345.0 | 332.7 | 344.2 |
| Chayote | 351.0 | 380.4 | 353.0 |
| Legumes |  |  |  |
| Tempeh | 658.8 | 176.9 | 625.0 |
| Tofu | 468.5 | 36.6 | 438.1 |
| Fruit and derivative products |  |  |  |
| Water apple | 237.7 | 166.0 | 232.7 |
| Banana | 179.1 | 249.6 | 184.1 |
| Guava | 168.9 | 253.2 | 174.9 |
| Bakery products |  |  |  |
| Bread | 591.3 | 275.3 | 569.1 |
| Cake | 316.0 | 38.4 | 296.5 |
| Biscuit | 54.3 | 61.5 | 54.8 |
| Spices |  |  |  |
| Spring onion | 164.9 | 83.2 | 159.1 |
| Chili and tomato sauce | 130.6 | 43.8 | 124.5 |
| Homemade chili sauce | 129.3 | 25.0 | 122.0 |
| Chili | 84.1 | 60.7 | 82.4 |
| Mixed foods/snacks |  |  |  |
| Vegetables fritter | 193.8 | 10.3 | 180.9 |
| Crackers | 135.4 | 12.7 | 126.7 |
| Fish and fishery products |  |  |  |
| Fish | 30.4 | 36.1 | 30.8 |
| Salted fish | 37.6 | 86.4 | 41.1 |
| Meat and meat products |  |  |  |
| Chicken | 76.0 | 3.8 | 70.9 |
| Beef | 55.2 | 3.2 | 51.6 |
| Eggs and dairy products |  |  |  |
| Egg | 98.5 | 3.2 | 91.8 |
| Drinks |  |  |  |
| Coffee | 310.1 | 71.7 | 293.4 |
| Tea | 197.1 | 159.1 | 194.4 |
