# Supplementary material for: The quantity and composition of household food waste: Implications for policy
Source: PLoS One. 2024 Jun 12;19(6):e0305087. doi: 10.1371/journal.pone.0305087 (PMC11168659; doi:10.1371/journal.pone.0305087)
Supplement: Attachment — Submitted filename: PONE-D-24-03754_ reviewer.pdf [file pone.0305087.s004.pdf]

PLOS ONE

The quantity and composition of household food waste: implications for policy

--Manuscript Draft--

**Manuscript Number:**	PONE-D-24-03754
**Article Type:**	Research Article
**Full Title:**	The quantity and composition of household food waste: implications for policy
**Short Title:**	Household food waste and implications for policy
**Corresponding Author:**	Rian Diana<br>Universitas Airlangga<br>Surabaya, East Java INDONESIA
**Keywords:**	Food Waste; drink waste; household; rural; urban; waste composition analysis; sustainable consumption
**Abstract:**	This study aimed to analyse the disparities in the quantity, composition, and drivers of household food waste between urban and rural areas. Household food waste was assessed through waste compositional analysis for food and diaries for beverages. This cross-sectional study included 215 households in Bogor Regency, Indonesia. Comparisons between the two areas were performed using an independent t-test. The average of household food waste in Bogor Regency was 77 kg/cap/year (edible 37.7%, inedible 62.3%). Household food waste was higher in urban areas (79.4 kg/cap/year) than in rural areas (45.8 kg/cap/year) (p<0.001). Cereals, tubers and their derivatives (especially rice) and vegetables were the major contributors to edible food waste, whereas fruits were the main contributors to inedible food waste in both areas. Food waste drivers were spoilage/staleness/moldiness, changes in texture, short shelf life, cooking too much, and plate leftovers. Understanding the quantity, composition, and drivers of household food waste is pivotal for developing effective awareness campaigns and fostering behavioral changes to prevent household food waste.
**Order of Authors:**	Drajat Martianto, Dr.
	Rian Diana
	Yayuk Farida Farida Baliwati
	Dadang Sukandar
	Agung Hendriadi
**Additional Information:**	
**Question**	**Response**
**Financial Disclosure**<br><br>Enter a financial disclosure statement that describes the sources of funding for the work included in this submission. Review the submission guidelines for detailed requirements. View published research articles from *PLOS ONE* for specific examples.<br><br><br>This statement is required for submission and **will appear in the published article** if the submission is accepted. Please make sure it is accurate.	Yes

Powered by Editorial Manager® and ProduXion Manager® from Aries Systems Corporation

**Funded studies**<br>Enter a statement with the following details:<br>• Initials of the authors who received each award<br>• Grant numbers awarded to each author<br>• The full name of each funder<br>• URL of each funder website<br>• Did the sponsors or funders play any role in the study design, data collection and analysis, decision to publish, or preparation of the manuscript?<br><br>Did you receive funding for this work?	
Please add funding details.<br>as follow-up to "**Financial Disclosure**<br><br>Enter a financial disclosure statement that describes the sources of funding for the work included in this submission. Review the submission guidelines for detailed requirements. View published research articles from *PLOS ONE* for specific examples.<br><br>This statement is required for submission and **will appear in the published article** if the submission is accepted. Please make sure it is accurate.<br><br>**Funded studies**<br>Enter a statement with the following details:<br>• Initials of the authors who received each award<br>• Grant numbers awarded to each author<br>• The full name of each funder<br>• URL of each funder website<br>• Did the sponsors or funders play any role in the study design, data collection and analysis, decision to publish, or preparation of the manuscript?<br><br>Did you receive funding for this work?"	The authors thank the Neys-van Hoogstraten Foundation and IPB University [Rian Diana, grant numbers: 01 /NHF-IPB I 2022] for funding this study. The funders did not involve in the study design, data collection, data analysis and preparation of the manuscript or decision to publish.
Please select the country of your main research funder (please select carefully as in some cases this is used in fee calculation).	INDONESIA - ID

as follow-up to "**Financial Disclosure**<br><br>Enter a financial disclosure statement that describes the sources of funding for the work included in this submission. Review the submission guidelines for detailed requirements. View published research articles from *PLOS ONE* for specific examples.<br><br>This statement is required for submission and **will appear in the published article** if the submission is accepted. Please make sure it is accurate.<br><br>**Funded studies**<br>Enter a statement with the following details:<br>• Initials of the authors who received each award<br>• Grant numbers awarded to each author<br>• The full name of each funder<br>• URL of each funder website<br>• Did the sponsors or funders play any role in the study design, data collection and analysis, decision to publish, or preparation of the manuscript?<br><br>Did you receive funding for this work?"	
**Competing Interests**<br><br>Use the instructions below to enter a competing interest statement for this submission. On behalf of all authors, disclose any competing interests that could be perceived to bias this work—acknowledging all financial support and any other relevant financial or non-financial competing interests.<br><br>This statement is required for submission and **will appear in the published article** if the submission is accepted. Please make sure it is accurate and that any funding sources listed in your Funding Information later in the submission form are also declared in your Financial Disclosure statement.	The authors declare no conflict of interest for this publication.

View published research articles from *PLOS ONE* for specific examples.<br><br>**NO authors have competing interests**<br><br>Enter: *The authors have declared that no competing interests exist.*<br><br>**Authors with competing interests**<br><br>Enter competing interest details beginning with this statement:<br><br>*I have read the journal's policy and the authors of this manuscript have the following competing interests: [insert competing interests here]*<br><br>* typeset	
**Ethics Statement**<br><br>Enter an ethics statement for this submission. This statement is required if the study involved:<br><br>• Human participants<br>• Human specimens or tissue<br>• Vertebrate animals or cephalopods<br>• Vertebrate embryos or tissues<br>• Field research<br><br>Write "N/A" if the submission does not require an ethics statement.<br><br>General guidance is provided below. Consult the submission guidelines for detailed instructions. **Make sure that all information entered here is included in the Methods section of the manuscript.**	This study was approved by the Human Research Ethics Committee of Bogor Agricultural University (number 747/IT3.KEPMSM-IPB/SK/2022). Participants were fully informed of the study's objectives and benefits, and were provided with the right to withdraw at any time without consequences. Written informed consent was obtained from all participants prior to the interview.

**Format for specific study types**<br><br>**Human Subject Research (involving human participants and/or tissue)**<br>• Give the name of the institutional review board or ethics committee that approved the study<br>• Include the approval number and/or a statement indicating approval of this research<br>• Indicate the form of consent obtained (written/oral) or the reason that consent was not obtained (e.g. the data were analyzed anonymously)<br><br>**Animal Research (involving vertebrate animals, embryos or tissues)**<br>• Provide the name of the Institutional Animal Care and Use Committee (IACUC) or other relevant ethics board that reviewed the study protocol, and indicate whether they approved this research or granted a formal waiver of ethical approval<br>• Include an approval number if one was obtained<br>• If the study involved *non-human primates*, add *additional details* about animal welfare and steps taken to ameliorate suffering<br>• If anesthesia, euthanasia, or any kind of animal sacrifice is part of the study, include briefly which substances and/or methods were applied<br><br>**Field Research**<br><br>Include the following details if this study involves the collection of plant, animal, or other materials from a natural setting:<br>• Field permit number<br>• Name of the institution or relevant body that granted permission	
**Data Availability**<br><br>Authors are required to make all data underlying the findings described fully available, without restriction, and from the time of publication. PLOS allows rare exceptions to address legal and ethical concerns. See the PLOS Data Policy and FAQ for detailed information.	No - some restrictions will apply

Powered by Editorial Manager® and ProduXion Manager® from Aries Systems Corporation

A Data Availability Statement describing where the data can be found is required at submission. Your answers to this question constitute the Data Availability Statement and **will be published in the article**, if accepted.<br><br>**Important:** Stating 'data available on request from the author' is not sufficient. If your data are only available upon request, select 'No' for the first question and explain your exceptional situation in the text box.<br><br>Do the authors confirm that all data underlying the findings described in their manuscript are fully available without restriction?	
**Describe where the data may be found in full sentences. If you are copying our sample text, replace any instances of XXX with the appropriate details.**<br><br>• If the data are **held or will be held in a public repository**, include URLs, accession numbers or DOIs. If this information will only be available after acceptance, indicate this by ticking the box below. For example: *All XXX files are available from the XXX database (accession number(s) XXX, XXX.).*<br>• If the data are all contained **within the manuscript and/or Supporting Information files**, enter the following: *All relevant data are within the manuscript and its Supporting Information files.*<br>• If neither of these applies but you are able to provide **details of access elsewhere**, with or without limitations, please do so. For example:<br><br>*Data cannot be shared publicly because of [XXX]. Data are available from the XXX Institutional Data Access / Ethics Committee (contact via XXX) for researchers who meet the criteria for access to confidential data.*<br><br>*The data underlying the results presented in the study are available from (include the name of the third party*	All relevant data are within the manuscript and its Supporting Information files.

and contact information or URL).<br>• This text is appropriate if the data are owned by a third party and authors do not have permission to share the data.<br><br>* typeset	
Additional data availability information:	

Powered by Editorial Manager® and ProduXion Manager® from Aries Systems Corporation

Manuscript

Click here to access/download;Manuscript;QUANTITY AND COMPOSITION OF HOUSEHOLD FOOD WASTE.docx

The quantity and composition of household food waste: implications for policy

Drajat Martianto[1], Rian Diana[2,3]*, Yayuk Farida Baliwati[1], Dadang Sukandar[1], Agung Hendriadi[4]

[1]Department of Community Nutrition, Faculty of Human Ecology, IPB University, Bogor, Indonesia.

[2]Department of Nutrition, Faculty of Public Health, Universitas Airlangga, Surabaya, Indonesia.

[3]Program of Nutrition Science, Graduate School, IPB University, Bogor, Indonesia.

[4]Agroindustry Research Center, National Research and Innovation Agency, Jakarta, Indonesia

*Corresponding author

Email: rian.diana@fkm.unair.ac.id

*All authors contributed equally to this work

Abstract

This study aimed to analyse the disparities in the quantity, composition, and drivers of household food waste between urban and rural areas. Household food waste was assessed through waste compositional analysis for food and diaries for beverages. This cross-sectional study included 215 households in Bogor Regency, Indonesia. Comparisons between the two areas were performed using an independent t-test. The average of household food waste in Bogor Regency was 77 kg/cap/year (edible 37.7%, inedible 62.3%). Household food waste was higher in urban areas (79.4 kg/cap/year) than in rural areas (45.8 kg/cap/year) ($p<0.001$). Cereals, tubers and their derivatives (especially rice) and vegetables were the major contributors to edible food waste, whereas fruits were the main contributors to inedible food waste in both areas. Food waste drivers were spoilage/staleness/moldiness, changes in texture, short shelf life, cooking too much, and plate leftovers. Understanding the quantity, composition, and drivers of household food waste is pivotal for developing effective awareness campaigns and fostering behavioral changes to prevent household food waste.

Keywords. food waste, drink waste, household, rural, urban, waste composition analysis

Introduction

Food loss and waste refer to a reduction in both the quantity and quality of food intended for human consumption along the food supply chain (1). Food waste (FW) occurs in retail (e.g., supermarkets and traditional markets), food services (e.g., restaurants, catering, canteens, and home consumption), households, and individuals (2–6).

The United Nations Environment Programme (UNEP) estimated that 17% of the food produced globally was discarded or not consumed. In 2019, the global average FW reached 121

kg/capita/year, equivalent to 931 million tons of food discarded at retail and consumer levels. Furthermore, households accounted for 61% of the total FW, while food services and retail contributed 26% and 13%, respectively (7).

Indonesia is the fourth-most populous country in the world. A food loss and waste study in Indonesia showed that in the last 20 years (2000-2019) the FW trend has increased from 39% to 55%, equivalent to 5-19 million tons per year. As much as 80% of FW came from households, 44% of which was edible food (8). However, there is a lack of detailed data on the characteristics of FW in Indonesia using direct measurement. Halving global FW per capita at the retail and consumer levels is one of the targets of the SDGs 12. To achieve this target, it is necessary to obtain accurate FW measurements to identify the contributing factors.

Studies related to FW at the household level have been conducted in many countries, especially developed countries in Europe (9–14), America (15,16), Canada (17,18), and China (19). However, a disproportionate number of studies have been conducted in urban areas compared to rural areas (7). Moreover, studies on FW in middle-income countries, particularly Southeast Asia, are limited. A systematic review revealed that no study in Southeast Asia has compared the quantity and composition of FW in urban and rural areas using direct measurements (20). To address this gap, the current study aimed to analyse the differences in household FW between urban and rural areas.

Methods

Household food waste measurements were conducted from 4-30 October 2022 using two methods: the waste compositional analysis (WCA) for solid waste (food) and diaries for beverages. The WCA method was performed for eight consecutive days in accordance with the Indonesian National Standard (SNI) 19-3964-1994 for the collection and measurement of samples of urban

waste generation and composition. Food waste from home trash bins was collected daily, sorted, and weighed per food type using a QME brand digital scale (QM-5140), with a precision of up to 2 g. This method is suitable for mixed solid waste, such as households (21,22), and its accuracy is high because of direct weighing and waste sorting by type and composition, eliminating the possibility of under-reporting (21).

The diary method was used to quantify drink waste, which was carried out over a seven-day period. Household members were requested to record the type and quantity of discarded beverages as well as the reason for discarding them. However, this method has several limitations, such as participant forgetting to record, unrecorded beverages, inaccurate estimation of discarded drink quantities, and changes in behavior during the recording period (23,24). To mitigate these limitations, clear instructions and a well-designed recording system were provided (24). Drink waste is typically disposed through a sink, making it impossible to use the WCA method. In this study, participants were instructed to record discarded beverages daily, and were provided with a measuring cup (100 ml) to measure the quantity of drinking waste.

The sample size was determined in accordance with SNI 19-3964-1994, which considered the population size, number of households, and average number of household members in the two selected sub-districts. A total of 215 households were selected for the FW measurements: 150 in urban areas and 65 in rural areas. Multistage random sampling was employed to select the households. This study was conducted in two areas of Bogor Regency, Indonesia, between October and September 2022. The Cibinong Sub-district was selected as the urban area, as it is the largest population center in the regency, and 100% of its area has urban characteristics. The Sukajaya sub-district was chosen as a rural area because 63.6% of its area has rural characteristics.

The materials and tools used to measure FW using the WCA method were plastic bags, digital scales 0-400 kg, gloves, and recording forms. Diary form-filling guidelines, pens/pencils, and measuring cups were used to measure the diary method.

FW was calculated by weighting the number of households in each neighborhood and the number of family members in each household. An Independent sample t-test was conducted using IBM SPSS 21 to evaluate the differences in the quantity of FW between rural and urban areas.

To calculate the average FW in Bogor Regency in kg per capita per year, FW data were extrapolated, and the average FW generation data for urban and rural households were utilized. This method was adapted from the National Zero Waste Council, Canada (25). The total population (per village) of Bogor Regency at the end of 2022 was obtained from the Bogor Regency Population and Civil Registry Office. Additionally, extrapolation accounted for the total rural (390,047 people) and urban (5,037,021 people) populations in Bogor Regency (5,427,068 people).

This study was approved by the Human Research Ethics Committee of Bogor Agricultural University (number 747/IT3.KEPMSM-IPB/SK/2022). Participants were fully informed of the study's objectives and benefits, and were provided with the right to withdraw at any time without consequences. Written informed consent was obtained from all participants prior to the interview.

Results

Household Characteristics

The mean ages and educational levels of husbands and wives in urban areas were slightly higher than those in rural areas. Household income and expenditures in urban areas were almost double those in rural areas. (Table 1).

Table 1 Household characteristics (mean ± SD)

**Characteristics**	**Urban**	**Rural**
Husband's age (years)	45.7 ± 11.1	43.5 ± 10.9
Wife's age (years)	42.5 ± 10.8	39.4 ± 10.1
Husband's education (years)	10.8 ± 3.3	6.3 ± 2.0
Wife's education (years)	9.8 ± 3.5	5.8 ± 1.9
Number of household members (people)	4.0 ± 1.1	4.3 ± 1.3
Income (IDR/cap/month)	1,237,135 ± 774,449	486,903 ± 458,301
Household expenditure (IDR/cap/month)		
Food	509,096 ± 285,619	285,613 ± 119,398
Non-food	687,102 ± 443,620	392,396 ± 239,733
Total	1,196,198 ± 613,280	678,009 ± 296,817

Quantity and composition of FW

This study revealed that the average FW in urban areas (79.4/cap/year) was almost double that in rural areas (45.8 kg/cap/year). Additionally, the proportion of edible FW was higher in urban areas (38.2%) than in rural areas (23.4%). The findings indicated that urban areas generated more FW per capita than rural areas for edible, inedible, and total FW ($p<0.001$) (Table 2).

Urban areas generated a greater quantity of FW, both in terms of type and composition, than rural areas. An exception to this trend was observed in inedible FW from fruits and their derivative products as well as inedible FW from cereals, tubers, and their derivative products. Urban households produced a higher proportion of edible FW than their rural counterparts. In urban areas, the most commonly discarded foods were cereals, tubers, and their derivatives (17.9 kg/cap/year), whereas rural areas had a higher disposal of vegetables (4.6 kg/cap/year). Fruit and its derivative products were the most commonly discarded inedible FW in both urban and rural areas, at a rate of 29.9 kg/cap/year and 21.7 kg/cap/year, respectively. Additionally, over 70% of the edible FW in both regions consists primarily of cereals, tubers, their derivatives, and vegetables. Conversely, over 70% of inedible FW consisted of fruits, their derivatives, and vegetables (Table 2).

Table 2 Mean and standard deviation of FW quantity by type and food group in urban and rural areas (kg/cap/year)

**Food Groups**	**Urban**			**Rural**			**p**		
	Edible FW	***Inedible FW***	**Total FW**	***Edible FW***	***Inedible FW***	**Total FW**	***Edible* (urban-rural)**	***Inedible* (urban-rural)**	**Total (urban-rural)**
Cereals, tubers and their derivative products	17.9 ± 4.9	3 ± 0.8	20.9 ± 5.8	3.5 ± 1	3 ± 0.8	6.5 ± 1.8	<0.001	0.714	<0.001
Bakery products	1.1 ± 0.3	-	1.1 ± 0.3	0.4 ± 0.1	-	0.4 ± 0.1	<0.001	-	<0.001
Meat and meat products	0.2 ± 0.1	2.1 ± 0.6	2.3 ± 0.6	0 ± 0	1 ± 0.3	1 ± 0.3	<0.001	<0.001	<0.001
Fish and fishery products	0.2 ± 0.1	1.3 ± 0.3	1.5 ± 0.4	0.1 ± 0	0.2 ± 0	0.3 ± 0.1	<0.001	<0.001	<0.001
Eggs and dairy products	0.1 ± 0	2 ± 0.5	2.1 ± 0.6	0 ± 0	0.9 ± 0.2	0.9 ± 0.2	<0.001	<0.001	<0.001
Spices	0.7 ± 0.2	1.8 ± 0.5	2.5 ± 0.7	0.3 ± 0.1	0.8 ± 0.2	1.1 ± 0.3	<0.001	<0.001	<0.001
Legumes	1.3 ± 0.3	0.6 ± 0.2	1.9 ± 0.5	0.2 ± 0.1	0.9 ± 0.3	1.2 ± 0.3	<0.001	<0.001	<0.001
Vegetables	6.2 ± 1.7	8.3 ± 2.3	14.4 ± 4	4.6 ± 1.3	6.6 ± 1.8	11.2 ± 3.1	<0.001	<0.001	<0.001
Fruit and derivative products	1.1 ± 0.3	29.9 ± 8.3	31 ± 8.6	1 ± 0.3	21.7 ± 6	22.7 ± 6.3	0.424	<0.001	<0.001
Candy and chocolate	0 ± 0	-	0 ± 0	0 ± 0	-	0 ± 0	<0.001	-	<0.001
Mixed foods/snacks	0.9 ± 0.2	-	0.9 ± 0.2	0.2 ± 0.1	-	0.2 ± 0.1	<0.001	-	<0.001
Other foods	-	0.1 ± 0	0.1 ± 0	-	0 ± 0	0 ± 0	-	<0.001	<0.001
Drinks	0.7 ± 0.2	-	0.7 ± 0.2	0.3 ± 0.1	-	0.3 ± 0.1	<0.001	-	<0.001
Total	30.3 ± 8.4	49.1 ± 13.5	79.4 ± 21.9	10.7 ± 3	35.1 ± 9.7	45.8 ± 12.6	<0.001	<0.001	<0.001

The most discarded edible waste per capita annually was rice (14.2 kg), carrots (0.9 kg), Chinese cabbage (0.7 kg), cabbage (0.6 kg), tempeh (0.6 kg), tofu (0.4 kg), guava (0.2 kg), and water apples (0.2 kg). Among the beverages, coffee (0.3 kg) and tea (0.2 kg) had the highest annual per capita waste, with animal food per capita being less than 0.1 kg (supplementary file).

Table 3 reveals that the average household FW in Bogor District is 77 kg/cap/year, consisting of 37.7% edible FW (29 kg/cap/year) and 62.3% inedible FW (48 kg/cap/year). Edible FW was mainly composed of cereals, tubers, and their derivatives at 16.9 kg/cap/year (mainly rice), and vegetables at 9.6 kg/cap/year. Meanwhile, inedible FW was dominated by fruits and their derivative products, accounting for 21.7 kg/cap/year (mainly bananas and mangoes).

Table 3 Mean and proportion of FW quantity by type and food group in Bogor District (kg/cap/year)

**Food Groups**	***Edible FW***	**%**	***Inedible FW***	**%**	**Total FW**	**%**
Cereals, tubers and their derivative products	16.9	58.3	3.0	6.3	19.9	25.8
Bakery products	1.1	3.8	0	0.0	1.1	1.4
Meat and meat products	0.2	0.7	2.0	4.2	2.2	2.9
Fish and fishery products	0.2	0.7	1.2	2.5	1.4	1.8
Eggs and dairy products	0.1	0.3	1.9	4.0	2.0	2.6
Spices	0.7	2.4	1.7	3.5	2.4	3.1
Legumes	1.2	4.1	0.6	1.3	1.8	2.3
Vegetables	6.1	21.0	8.1	16.9	14.2	18.4
Fruit and derivative products	1.1	3.8	29.4	61.3	30.5	39.6
Candy and chocolate	0	0.0	0	0.0	0	0.0

Mixed foods/snacks	0.8	2.8	0	0.0	0.8	1.0
Other foods	0		0.1	0.2	0.1	0.1
Drinks	0.6	2.1	0		0.6	0.8
Total	29	100.0	48	100.0	77	100.0

Reasons for throwing away food

Five primary reasons were identified for discarding food: spoilage/staleness/moldiness, changes in texture, short shelf life, cooking too much, and plate leftovers. Sensory characteristics, such as staleness or moldiness and changes in texture, were the main reasons for throwing away rice and vegetables. The fruits were discarded because of moldiness, bruises, and changes in texture. Both animal- and plant-based foods have been discarded for various reasons. Dairy products were often discarded because they had been forgotten in the refrigerator and remained unconsumed. Frequent reasons for discarding meat, fish, and eggs were mold or mildew, and textural changes. Meanwhile, tofu and tempeh were discarded due to cooking too much, changes in texture, and unconsumed. Bread was discarded when it was moldy or exceeded its best-before date. Inappropriate storage temperature and sensory changes (staleness or moldiness, and texture changes) were the main reasons for discarding packaged food (see Table 4). The reasons reported by the respondents in both urban and rural areas were almost identical for all types of food.

Table 4 Number of respondents by FW drivers

**Food**	**Spoil/ Stale/ Moldy**	**Cooking too much**	**Short shelf life**	**Changes texture**	**Plate leftovers**	**Forgotten**	**Others**
Rice	52	53	24	56	57	3	21
Vegetables	63	48	60	9	27	8	37

Fruits	32	1	3	15		5	9
Dairy products	4		4		5	10	9
Meat, fish, and eggs	14	6	6	12	6	1	15
Tempeh and tofu	6	25	4	9	9	7	12
Breas	31	1	1	1		2	9
Packaged foods	5			5	1	3	19
Cooked foods	11	2	11	4	1		15
Total	218	136	113	111	106	39	146

Discussion

The average FW found in this study (77 kg/cap/year) was comparable to the average FW in upper-middle-income (76 kg/cap/year) and high-income (79 kg/cap/year) countries (7). In contrast, edible FW in upper-middle-income and high-income countries is higher, ranging from 50-65% (10–13,26) compared with the 37.7% found in this study, which is a lower-middle-income country. The discarded food groups across countries displayed a similar composition, with staple foods, fruits, and vegetables being the main contributors to the total FW. This study aligns with previous research conducted in high-income countries, where staple diets consisting of rice (19) or bread (11,27–29).

This study revealed that the average FW in urban areas was twice as high as that in rural areas, which is consistent with previous research conducted in lower, middle, and high-income countries (30,31). The quantity of FW in urban areas (79.4 kg/cap/year; 38.2% edible) exceeded that in rural areas (45.8 kg/cap/year; 23.4% edibl[illegible]nevertheless, the composition and reasons for discarding food were similar between the two regions. The most commonly discarded edible FW in both

urban and rural areas consists of cereals, tubers and their derivatives, vegetables, and legumes. The main contributors to inedible food waste were fruits and their derivatives, along with vegetables.

Various reasons underlie food waste, depending on the type of food. Information on food storage, including techniques to extend the shelf life of food and proper food storage practices, is applicable to all types of food. Encouraging behavioral changes in cooking and eating habits is crucial to preventing and reducing food waste, especially for foods such as rice, vegetables, plant-based foods (tofu and tempeh), and cooked foods. Utilizing data-related information on food packaging can help prevent wastage of bread and packaged foods.

The proportion of inedible FW was higher than that of edible FW in both the urban and rural areas. Urban households were more likely to discard edible FW than rural households (38.2% and 23.4 %, respectively). This implies that urban households throw away more food suitable for consumption. Consistent with studies in the European Union and Lebanon, this study found that the quantity of household FW in urban areas was higher than that in rural areas (30,31). The differences in household income and food expenditure between urban and rural areas may have influenced the results of this study. Residents in urban areas have higher incomes but a lower proportion of food expenditure than those living in rural areas, leading to a higher diversity in food consumption (32). The higher the diet quality, the greater the FW, as individuals with a high dietary quality consume greater quantities of vegetables and fruits, which are the most discarded food waste (33).

The different generations of household FW in rural and urban areas can be caused by disparities in food systems and the utilization or management of food waste. Self-produced food sources are more common in rural areas than in urban areas. Conversely, urban households tend to purchase

processed food, contributing more significantly to FW than rural areas (34). Furthermore, many rural households convert their food waste into compost or use it as feed for pets or livestock (35,36), thereby reducing the amount of FW disposed in bins (35).

Modern retail, restaurants, and fast-food outlets are more abundant in urban areas than in rural areas. The growth of modern retail can affect traditional retail, consumer preferences, purchasing behavior, and food accessibility (32). Urban residents tend to prefer quick and convenient food preparation, leading to food over-purchasing, high consumption of processed foods, and foods from outside the home (restaurants, fast food outlets, and street food) (34). This trend contributes to the generation of food waste (34,37).

Policy Implication

Policies aimed at preventing household food waste should cover both preventive measures and food waste management. Prevention involves actions taken before edible food is discarded, whereas management focuses on dealing with food that is no longer suitable for consumption or has already been discarded. Prevention strategies can be implemented through awareness campaigns and initiatives to encourage behavioral changes. On the other hand, managing food waste can involve practices such as reuse and recycling by converting it into feed, compost, and eco-enzymes.

Prevention and reduction strategies should focus on urban areas and food groups that contribute significantly to edible FW (such as cereals, tubers, and their derivatives, particularly rice, vegetables, and legumes). Prioritizing the reuse and recycling of inedible food waste, particularly from fruits and vegetables, is essential to prevent its disposal in landfills.

Behavioral changes that focus on prolonging the shelf life of food and practicing proper storage can be implemented for all types of foods. Adjusting cooking portions and eating habits can be particularly beneficial for rice, vegetables, plant-based proteins such as tofu and tempeh, and cooked foods. Understanding food packaging data can help minimize food waste, particularly for bread and packaged goods. Finally, reusing food waste for feed and composting inedible fruits and vegetables at home rather than sending them to landfills is an important step in reducing food waste.

Research limitation

This study was conducted using direct FW measurements (Waste Composition Analysis) over eight consecutive days, ensuring high accuracy through sorting and weighing, based on type and composition. However, this study only captured a single point in time and did not include seasonal data. Additionally, the study only measured FW disposed of in household bins and did not consider FW disposed of or given to pets, plants, or other disposal channels. Despite these limitations, this study found that over 82.8% of household food waste was disposed of in the bin, with only 16.7% discarding FW onto plants for composting.

Conclusion

The quantity of household FW differs significantly between the urban and rural areas. However, based on the similar FW compositions and underlying reasons, strategies for FW prevention and reduction can be applied uniformly across both regions. Disparities exist in FW drivers, primarily depending on the type of food rather than the location of the resident

Acknowledgements

The authors thank the Neys-van Hoogstraten Foundation and IPB University [Rian Diana, grant numbers: 01 /NHF-IPB I 2022] for funding this study. The funders did not involve in the study design, data collection, data analysis and preparation of the manuscript or decision to publish.

Competing Interest

The authors declare no conflict of interest for this publication.

References

1. Gustavsson J, Cederberg C, Sonesson U, van Otterdijk R, Meybeck A. Global Food Losses and Food Waste. Extent, Causes and Prevention [Internet]. Rome: FAO; 2011. Available from: https://www.fao.org/3/i2697e/i2697e.pdf

2. Schanes K, Dobernig K, Gözet B. Food Waste Matters - A Systematic Review of Household Food Waste Practices and Their Policy Implications. J Clean Prod [Internet]. 2018;182:978–91. Available from: https://doi.org/10.1016/j.jclepro.2018.02.030

3. Mulyana IJ, Gunawan I, Tamara V. Studi Eksploratif Manajemen Sampah Makanan pada Jaringan Toko Produk Pangan di Indonesia. Ind J Teknol dan Manaj Agroindustri. 2019;8(2):95–106.

4. Dhir A, Talwar S, Kaur P, Malibari A. Food waste in hospitality and food services: A systematic literature review and framework development approach. J Clean Prod [Internet]. 2020;270:122861. Available from: http://www.sciencedirect.com/science/article/pii/S0959652620329061

5. Teller C, Holweg C, Reiner G, Kotzab H. Retail store operations and food waste. J Clean Prod [Internet]. 2018;185:981–97. Available from: http://www.sciencedirect.com/science/article/pii/S0959652618306127

6. Ho KS, Chu LM. Characterization of food waste from different sources in Hong Kong. J Air Waste Manage Assoc [Internet]. 2019 Mar 4;69(3):277–88. Available from: https://doi.org/10.1080/10962247.2018.1526138

7. United Nations Environment Programme. Food Waste Index Report 2021 [Internet]. Nairobi: United Nations Environment Programme; 2021. Available from: https://www.unep.org/resources/report/unep-food-waste-index-report-2021

8. Ministry of National Development Planning/ Bappenas. Study Report Food Loss and Waste in Indonesia Supporting the Implementation of Circular Economy and Low Carbon Development [Internet]. Jakarta: Ministry of National Development Planning/ Bappenas; 2021. Available from: https://lcdi-indonesia.id/wp-content/uploads/2021/07/Report-Kajian-FLW-ENG.pdf

9. Bravi L, Francioni B, Murmura F, Savelli E. Factors affecting household food waste among young consumers and actions to prevent it. A comparison among UK, Spain and Italy. Resour Conserv Recycl. 2020;153(September 2019):104586.

10. Elimelech E, Ayalon O, Ert E. What gets measured gets managed: A new method of measuring household food waste. Waste Manag. 2018 Jun;76:68–81.

11. Hanssen OJ, Syversen F, Stø E. Edible food waste from Norwegian households—Detailed food waste composition analysis among households in two different regions in Norway. Resour Conserv Recycl [Internet]. 2016;109:146–54. Available from:

http://www.sciencedirect.com/science/article/pii/S0921344916300441

12. van Dooren C, Janmaat O, Snoek J, Schrijnen M. Measuring food waste in Dutch households: A synthesis of three studies. Waste Manag. 2019 Jul;94:153–64.

13. Edjabou ME, Petersen C, Scheutz C, Astrup TF. Food waste from Danish households: Generation and composition. Waste Manag. 2016 Jun;52:256–68.

14. De Laurentiis V, Corrado S, Sala S. Quantifying household waste of fresh fruit and vegetables in the EU. Waste Manag. 2018 Jul;77:238–51.

15. Spiker ML, Hiza HAB, Siddiqi SM, Neff RA. Wasted Food, Wasted Nutrients: Nutrient Loss from Wasted Food in the United States and Comparison to Gaps in Dietary Intake. J Acad Nutr Diet [Internet]. 2017;117(7):1031–1040.e22. Available from: http://www.sciencedirect.com/science/article/pii/S2212267217303258

16. Conrad Z, Niles MT, Neher DA, Roy ED, Tichenor NE, Jahns L. Relationship between food waste, diet quality, and environmental sustainability. PLoS One. 2018;13(4):e0195405.

17. Carroll N, Wallace A, Jewell K, Darlington G, Ma DWL, Duncan AM, et al. Association between diet quality and food waste in Canadian families: a cross-sectional study. Nutr J. 2020 Jun;19(1):54.

18. Van Der Werf P, Seabrook JA, Gilliland JA. The quantity of food waste in the garbage stream of southern Ontario, Canada households. PLoS One. 2018;13(6):e0198470.

19. Zhang H, Duan H, Andric JM, Song M, Yang B. Characterization of household food waste and strategies for its reduction: A Shenzhen City case study. Waste Manag.

2018;78:426–33.

20. Diana R, Martianto D, Baliwati YF, Sukandar D, Hendriadi A. Determinants of Household Food Waste in Southeast Asia: A Systematic Review. J Hunger Environ Nutr [Internet]. 2023; Available from: https://doi.org/10.1080/19320248.2023.2174060

21. FLW Protocol Steering Committee. Food Loss + Waste Protocol. Guidance in FLW Quantification Methods. Supplement to the Food Loss and Wate (FLW) Accountinng and Reporting Standard, Version 1.0. World Resorces Institute; 2016.

22. O'Connor C. Food Waste Index Update [Internet]. 2020 [cited 2020 Oct 29]. Available from: https://ec.europa.eu/food/sites/food/files/safety/docs/fw_eu-platform_20200615_flw_pres_09.pdf

23. Quested TE, Palmer G, Moreno LC, McDermott C, Schumacher K. Comparing diaries and waste compositional analysis for measuring food waste in the home. J Clean Prod [Internet]. 2020;262:121263. Available from: https://www.sciencedirect.com/science/article/pii/S095965262031310X

24. WRAP. Household Food and Drink Waste in the UK. Banbury: WRAP; 2009.

25. National Zero Waste Council. How to Measure Food Waste: A Guide for Measuring Food Waste from Household in Canada [Internet]. Vancouver: National Zero Waste Council,, Canada; 2018. Available from: http://www.nzwc.ca/Documents/LFHW_GuideToMeasuringFoodLossAndWaste.pdf#search=Appendix D%3A Extrapolation Methods

26. Parizeau K, von Massow M, Martin RC. Directly observing household food waste

generation using composition audits in a Canadian municipality. Waste Manag [Internet]. 2021;135:229–33. Available from: https://www.sciencedirect.com/science/article/pii/S0956053X21004827

27. Williams H, Lindström A, Trischler J, Wikström F, Rowe Z. Avoiding food becoming waste in households – The role of packaging in consumers' practices across different food categories. J Clean Prod [Internet]. 2020;265:121775. Available from: https://www.sciencedirect.com/science/article/pii/S0959652620318229

28. Djekic I, Miloradovic Z, Djekic S, Tomasevic I. Household food waste in Serbia – Attitudes, quantities and global warming potential. J Clean Prod [Internet]. 2019;229:44–52. Available from: https://www.sciencedirect.com/science/article/pii/S095965261931501X

29. Sosna D, Brunclíková L, Galeta P. Rescuing things: Food waste in the rural environment in the Czech Republic. J Clean Prod [Internet]. 2019;214:319–30. Available from: https://www.sciencedirect.com/science/article/pii/S0959652618339349

30. Secondi L, Principato L, Laureti T. Household food waste behaviour in EU-27 countries: A multilevel analysis. Food Policy. 2015;56:25–40.

31. Mattar L, Abiad MG, Chalak A, Diab M, Hassan H. Attitudes and behaviors shaping household food waste generation: Lessons from Lebanon. J Clean Prod. 2018;198:1219–23.

32. Proctor FJ, Berdegué JA. Food systems at the rural-urban interface. Santiago, Chile; 2016. (Working Paper series N° 194N° 194).

33. Brennan A, Browne S. Food waste and nutrition quality in the context of public health: A scoping review. Int J Environ Res Public Health. 2021;18(10):5379.

34. FAO. Dynamic Development, Shifting Demographics, Changing Diets. Bangkok: FAO; 2018.

35. Niles MT. Majority of Rural Residents Compost Food Waste: Policy and Waste Management Implications for Rural Regions. Front Sustain Food Syst. 2020;3(January):Article 123.

36. Chammas G, Yehya NA. Lebanese meal management practices and cultural constructions of food waste. Appetite [Internet]. 2020;155:104803. Available from: https://www.sciencedirect.com/science/article/pii/S0195666319313315

37. McCarthy B, Liu HB. Food waste and the "green" consumer. Australas Mark J. 2017;25(2):126–32.

Supplementary table

Click here to access/download
Supporting Information
Supplementary Table 1 frequent edible food waste.docx
