# Supplementary material for: The quantity and composition of household food waste: Implications for policy
Source: PLoS One. 2024 Jun 12;19(6):e0305087. doi: 10.1371/journal.pone.0305087 (PMC11168659; doi:10.1371/journal.pone.0305087)
Supplement: Attachment — Submitted filename: comments.docx [file pone.0305087.s003.docx]

This study focuses on analyzing the differences in the amount, types, and causes of household food waste between urban and rural areas. It was conducted in Bogor Regency, Indonesia, and involved 215 households. The researchers used waste compositional analysis for food and diaries for beverages to assess household food waste. However, it could benefit from more detailed explanations of the methodology, a deeper analysis of findings, and a more comprehensive presentation to provide a clearer picture of the research. Some of the issues which needs to be addressed before further process are the following:

**Abstract:**

1. This abstract need revision: Background and Gap (reason you are conducting this study in this area), are the main missing parts in this abstract.

**Introduction:**

2. The citation markers (e.g., "(1)", "(2-6)") disrupt the flow of the text and could be incorporated more smoothly. Please use a proper citation style.

3. The transition between discussing global food waste and focusing on Indonesia could be smoother. Consider using transitional phrases to connect these sections more effectively.

4. line 50, you said “Moreover, studies on FW in middle-income countries, particularly Southeast Asia, are limited.” However, you didn’t acknowledge the limitations of existing research. It's important to acknowledge the limitations of existing research, particularly the lack of detailed data on food waste in Indonesia. This could help set the stage for the current study and emphasize its importance.

You didn’t your study area and made all data underlying the findings in your manuscript fully available. Please do it.

**Methodology:**

**5.** While the study mentions the use of multistage random sampling, it does not elaborate on the specific details of how households were selected within each stage.

6. This approach introduces inconsistencies, potentially affecting data reliability. The diary method has limitations like forgetfulness and inaccurate estimations. I suggest to improve measurement techniques, you implement “waste audits”, where trained personnel sort and weigh household waste samples over an extended period. You can also use “Smart sensor technology”. This Smart sensor integrated into trash bins to automatically quantify and categorize waste, providing real-time data collection **without relying on self-reporting**.

7. The study mentions obtaining written informed consent from participants and approval from the Human Research Ethics Committee. However, it does not provide details on how privacy and confidentiality were ensured, especially given the sensitive nature of waste-related data.

8. The adaptation of the method from the National Zero Waste Council of Canada introduces a potential bias if the waste generation patterns and waste management practices in Indonesia differ significantly from those in Canada. This raises questions about the validity and applicability of the methodology to the local context.

**Result:**

9. Why can’t you present your result by figures? The figures presented in the comparison study could provide valuable insights into the differences and similarities between the urban and rural contexts.

10. Line 109. While statistical significance (p<0.001) is mentioned, it would be beneficial to provide confidence intervals or other measures of uncertainty to qualify the findings and indicate the robustness of the observed differences between urban and rural areas.

11. Line 135. Reasons for throwing away food:

Better by comparison: “why do urban areas generate more FW per capita? Are there specific consumption patterns, food distribution systems, or waste management practices that contribute to this disparity?”

**Discussion:**

12. The discussion does not provide a clear context for the study or its significance. It merely states the findings without explaining why the study was conducted or what implications these findings might have. It lacks depth in analysis and fails to offer comprehensive solutions to address the complex issue of food waste.

13. please cite your results (tables, figures) in this part

**Conclusion:**

14. This conclusion oversimplifies the complexities of FW generation and overlooks the importance of context-specific factors in determining effective prevention and reduction strategies. A more nuanced analysis taking into account the unique characteristics of each setting would provide a stronger foundation for developing targeted interventions to address FW in both urban and rural areas.
